# Adaptation to Fluctuating Temperatures in an RNA Virus Is Driven by the Most Stringent Selective Pressure

**DOI:** 10.1371/journal.pone.0100940

**Published:** 2014-06-25

**Authors:** María Arribas, Kirina Kubota, Laura Cabanillas, Ester Lázaro

**Affiliations:** 1 Centro de Astrobiología (INTA-CSIC), 28850 Torrejón de Ardoz, Madrid, Spain; 2 Facultad de Ciencias, Universidad Autónoma de Madrid, 28049 Cantoblanco, Madrid, Spain; University of Florida, United States of America

## Abstract

The frequency of change in the selective pressures is one of the main factors driving evolution. It is generally accepted that constant environments select specialist organisms whereas changing environments favour generalists. The particular outcome achieved in either case also depends on the relative strength of the selective pressures and on the fitness costs of mutations across environments. RNA viruses are characterized by their high genetic diversity, which provides fast adaptation to environmental changes and helps them evade most antiviral treatments. Therefore, the study of the adaptive possibilities of RNA viruses is highly relevant for both basic and applied research. In this study we have evolved an RNA virus, the bacteriophage Qβ, under three different temperatures that either were kept constant or alternated periodically. The populations obtained were analyzed at the phenotypic and the genotypic level to characterize the evolutionary process followed by the virus in each case and the amount of convergent genetic changes attained. Finally, we also investigated the influence of the pre-existent genetic diversity on adaptation to high temperature. The main conclusions that arise from our results are: i) under periodically changing temperature conditions, evolution of bacteriophage Qβ is driven by the most stringent selective pressure, ii) there is a high degree of evolutionary convergence between replicated populations and also among populations evolved at different temperatures, iii) there are mutations specific of a particular condition, and iv) adaptation to high temperatures in populations differing in their pre-existent genetic diversity takes place through the selection of a common set of mutations.

## Introduction

The frequency of change in the selective pressures together with the fitness effects of mutations across environments are major determinants of the evolutionary pathway followed by populations. When selective pressures remain constant for a long time, specialist organisms, which perform well in a particular environment and poorly elsewhere, are favoured [1]–[7]. In contrast to this, when the selective pressures change frequently, fitness costs of mutations across environments can limit adaptation [6], [8]–[10], promoting the selection of generalist organisms able to cope with a variety of conditions [8], [11]–[15]. Generalists frequently select genotypes containing mutations of beneficial effects under all the selective pressures that are to be encountered. Another possibility is to maintain as polymorphisms mutations advantageous in some environments but deleterious under alternative conditions. The existence of different targets of selection through time together with the high frequency of fitness trade-offs has led to the proposition that generalism could have an associated fitness cost [7], [11], [14], [16]–[18].

Many of the studies concerning the selection of generalism and specialism have been carried out through experimental evolution, which requires systems that evolve quickly and that allow to establish accurate correspondences between phenotypes and genotypes. Since RNA viruses are able to adapt in very short times to environmental changes [19], and also posses small genomes, they have been used oftenly [20]–[33]. The high adaptive capacity of RNA viruses lies on their elevated error rates that promote the generation of populations composed by a mutant spectrum with a very wide phenotypic and genetic heterogeneity [19], [34]. In these populations, mutants dominant under previous selective pressures can be kept as minority variants of the mutant spectrum and be rapidly selected when the virus is exposed again to the same condition. This reservoir of variants provides a molecular memory [35]–[37] which could be very useful during adaptation to fluctuating selective pressures. Typically, studies carried out with RNA viruses compare the results obtained when a selective pressure, commonly the virus host or some abiotic factor, either remains constant or alternates between different values. In most cases a pattern of specialization under constant conditions was found, which was occasionally accompanied by a cost in alternative environments [21], [22], [25], [26], [28], [30], [31]. Populations that alternated different environments in some cases presented a generalism-associated fitness cost [14], although in others they showed equal or superior improvement in each environment than the lineages evolved constantly in the same conditions [22], [24], [27]–[29], [32], [33].

In this study we have propagated a heterogeneous population of an RNA virus, the bacteriophage Qβ, at its optimal growth temperature (37°C) and at two new temperatures, one above the optimal value (43°C) and another one below (30°C). To compare the virus populations in constant versus deterministically fluctuating environments, we carried out evolution experiments in which the temperature was kept invariable throughout the process, together with other ones in which the temperature alternated periodically among the three values assayed, using two patterns of change. The analysis of the evolved populations allowed us to determine the evolutionary pathway followed by the virus under the different propagation conditions. We also analyzed the genomic changes candidate to be responsible for adaptation and the degree of evolutionary convergence between independent replicated experiments as well as among populations evolving under different conditions, including changing environments. Finally, we investigated whether the genetic changes that allow adaptation to a particular condition (in this case, high temperature) were the same or not in populations differing in their initial genetic diversity.

## Materials and Methods

### Viruses and bacteria: Standard procedures for infection

Bacteriophage Qβ was routinely propagated by infecting log-phase cultures of *Escherichia coli*, strain Hfr (Hayes) in NB medium (8 g/l Nutrient Broth from Merck and 5 g/l NaCl). The virus, which was provided by Prof. C. K. Biebricher, was adapted to replicate in liquid cultures in our laboratory by infecting 1 liter of an exponential phase *E. coli* culture at a multiplicity of infection (moi) of 1 plaque forming unit (pfu) per bacterium. The culture was incubated at 37°C with aeration during 90 min and subsequently an aliquot was used to infect another fresh exponential culture of *E. coli* at the same moi. This process was repeated for a total of 5 serial transfers [38]. The lysate at the fifth transfer was clarified by incubating with a few drops of chloroform for 30 min at room temperature with aeration, after which it was centrifuged for 40 min at 8000×g. Phages from the supernatant were purified by precipitation with 6% polyethylene glycol 6000 and differential sedimentation [38]. The virus population obtained in this way was called Qβ_0_, which was used as ancestor population for most of the evolution experiments described in this paper. We chose to use a heterogeneous population instead of a clone to better reproduce the action of natural selection in natural virus populations, which normally show high diversity.

Infections in liquid medium were always carried out using fresh exponential phase *E. coli* cultures (with an optical density at 550 nm between 0.6 and 0.8) that were infected with the virus at the moi indicated in each experiment. After 2 h of incubation at 37°C with aeration, cultures were treated with 1/20 vol of chloroform for 15 min at 37°C with shaking (300 rpm). Virus supernatants were harvested upon centrifugation at 13000×g for 10 min and maintained at 4°C for short-term use (less than 15 days) or at −80°C for long-term storage. Virus titres were determined by plaque assay and expressed as the number of pfu per ml of the phage suspension.

### Evolution experiments

**Figure 1:**
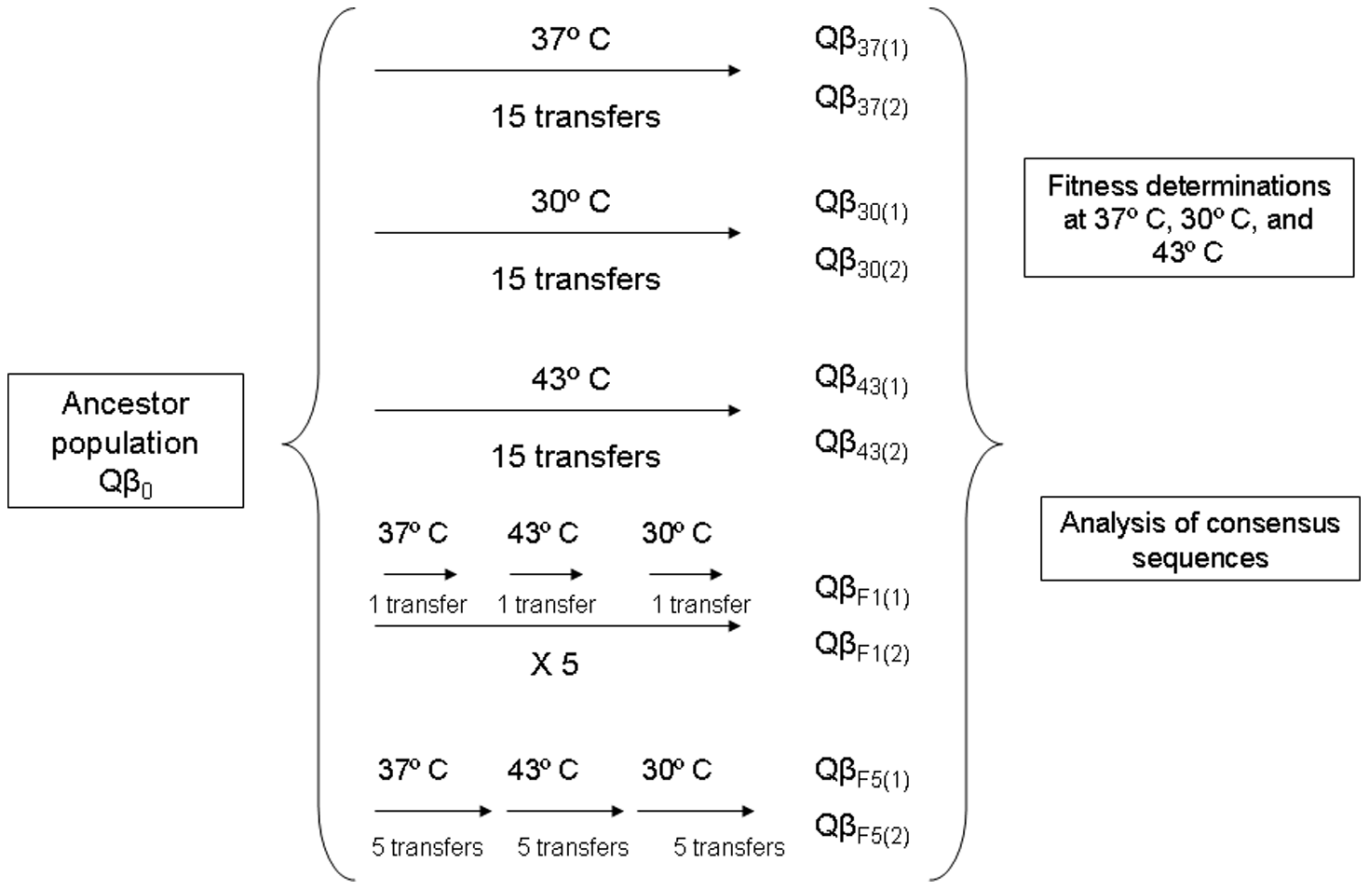
Description of the evolution experiment designed to study the adaptation of bacteriophage Qβ to constant or deterministically fluctuating temperatures. The same virus population (Qβ_0_) was used to found 5 replicated evolutionary lineages that were evolved in parallel during 15 transfers either at constant (populations Qβ_37,_ Qβ_30,_ and Qβ_43_)_,_ or fluctuating temperatures (populations Qβ_F1_, and Qβ_F5_) under the conditions described in Materials and Methods. Replicate populations were labelled with the subscripts (1) and (2). Each evolved population was analyzed at the phenotypic level (determination of fitness at 37°C, 30°C, and 43°C), and at the genotypic level (determination of the consensus sequence from nucleotide 250 to 4180).

Adaptation to 30°C and 37°C was carried out by infecting 10^8^ bacteria in exponential phase with 10^6^ pfu of the population Qβ_0_ in a final volume of 1 ml in 10 ml polypropylene tubes. Since virus growing at 43°C was strongly inhibited, adaptation to this temperature was carried out under conditions that yielded higher virus titres (10^9^ bacteria infected with 10^7^ pfu in a final volume of 10 ml in 50 ml flasks). All experiments were carried out in duplicate. After 2 h of incubation at 37°C with aeration, the virus supernatants were collected as described above and the virus titres were estimated by plaque assay. The amount of phage suspensions containing 10^6^ pfu (cultures performed at 30°C and 37°C) or 10^7^ pfu (cultures carried out at 43°C) was used to infect a fresh *E. coli* culture. The procedure was repeated for a total of 15 transfers under constant conditions to obtain the virus populations Qβ_37_, Qβ_30_, and Qβ_43_ (Fig. 1). The approximate number of replication rounds experienced by the virus during the 15 transfers were 235 (Qβ_37_), 200 (Qβ_30_), and 120 (Qβ_43_) Adaptation to deterministically fluctuating temperatures took place by alternating 37°C, 43°C, and 30°C in this order, with the temperature change occurring either every transfer (populations Qβ_F1_) or every five transfers (populations Qβ_F5_) (Fig. 1). The culture conditions for each temperature were the same as described for experiments carried out at constant temperatures. In all cases replicate lineages were distinguished with numbers 1 and 2.

**Figure 2:**
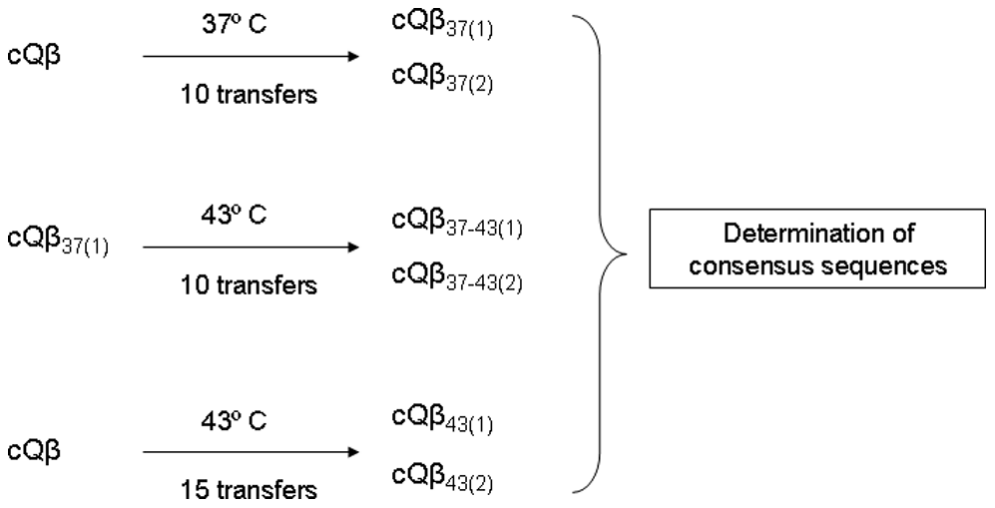
Description of the evolution experiment designed to study the influence of the pre-existent genetic diversity on the adaptation of bacteriophage Qβ to high temperature. The clonal population of the virus, cQβ, obtained upon expression of the plasmid pBRT7Qβ (see Materials and Methods), was propagated at 37°C during 10 serial transfers to yield populations cQβ_37(1)_ and cQβ_37(2)_. Population cQβ_37(1)_ was subjected to 10 additional transfers at 43°C, to yield populations cQβ_37-43(1)_ and cQβ_37-43(2)._ The virus cQβ was also adapted to 43°C without previous optimization at 37°C. Adaptation took place through 15 serial transfers to render populations cQβ_43(1)_ and cQβ_43(2)_. All evolved populations were characterized at the genotypic level through their consensus sequences (from nucleotide 250 to 4180).

Experiments designed to study adaptation in populations differing in their pre-existent genetic diversity (Fig. 2) were carried out using as ancestor population a clonal population of bacteriophage Qβ (cQβ). The clonal population was obtained upon expression of the plasmid pBRT7Qβ which contains a cDNA of the wild type virus cloned in the plasmid pBR322 [39]. Briefly, we transformed *E. coli* DH5-α with the plasmid pBRT7Qβ, and used the supernatant from an overnight culture of a transformed colony to infect a lawn of *E. coli* Hfr in semisolid agar. A lytic plaque was isolated, and the viruses contained on it were extracted and adapted to replicate at 37°C or 43°C as described above (Fig. 2).

### Fitness determinations

Growth rate values at 37°C, 30°C, or 43°C were used as a surrogate of fitness at each temperature. All the determinations were carried out by using liquid cultures that contained 10^8^ bacteria which were inoculated with 10^4^ pfu of the virus population indicated in a final volume of 1 ml. After two hours at 37°C with aeration, the virus supernatants were collected as described above and titrated to estimate the virus yield. Preliminary assays showed that bacteriophage Qβ grew exponentially under these conditions. Each determination was carried out in triplicate, and fitness was expressed as the log_2_ [(virus_end_ – virus_0_)/virus_0_], where virus_0_ was the initial input of virus in each experiment and virus_end_ was the number of pfu obtained at the end of the experiment.

### RNA extraction, cDNA synthesis, PCR amplification and nucleotide sequencing

Virus RNA was prepared following standard procedures [38], [40]. RNAs were amplified by RT-PCR using Avian Myeloblastosis Virus RT (Promega) and Expand High Fidelity DNA polymerase (Roche). The cDNAs were purified with a Qiagen purification kit and subjected to cycle sequencing with Big Dye Chemistry (Applied Biosystems; Perkin Elmer). The following pairs of oligonucleotide primers were used for RT-PCR: 5′CGAATCTTCCGACACGCATCC3′ and 5′AAACGGTAACACGCTTCTCCAG3′ to amplify from nucleotide position 150 to 1497; 5′CTCAATCCGCGTGGGGTAAATCC3′ and 5′CAGAAAATCGGCAGTGACGCAACA3′ to amplify from nucleotide position 1407 to 2817; and 5′GTGCCATACCGTTTGACT3′with 5′GATCCCCCTCTCACTCGT3′ to amplify from nucleotide position 2254 to 4195. Sequences were aligned with the consensus sequence of the wild type phage with Clustal W. Mutations relative to the consensus sequence were identified using the program BioEdit. Nucleotides were numbered according to the sequence of the cDNA of bacteriophage Qβ cloned in the plasmid pBR322 [39].

### Statistics

The statistical significance of the fitness differences between the ancestor and the evolved populations was evaluated through the Student'*t* test. A nested ANOVA, including the temperature of evolution as main factor and the triplicate measures of fitness as random variables, was performed for each temperature at which fitness was determined. A two-way ANOVA was carried out to compare fluctuating populations with those evolved under each constant temperature condition. The factors considered were the temperature of evolution and the temperature of the fitness assay. Each replicate population was characterized by three fitness values, each one corresponding to the average of three determinations carried out at each temperature. Calculations were carried out using Mathematica 5 (Wolfram Research)

## Results

### Evolution of bacteriophage Qβ at constant temperatures

**Figure 3:**
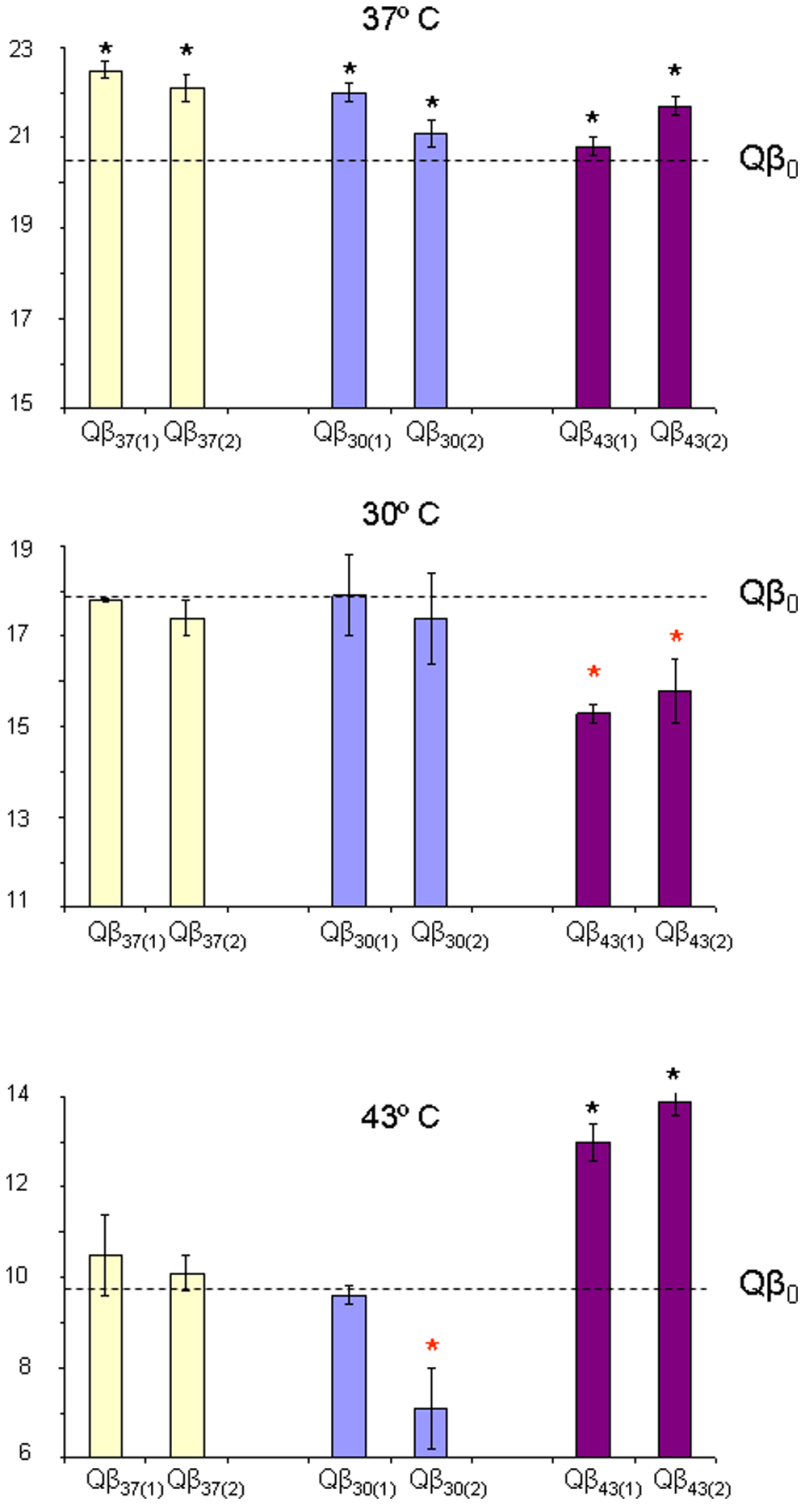
Fitness values of bacteriophage Qβ populations evolved at constant temperatures. Fitness values at 37°C (upper panel), 30°C (medium panel), and 43°C (lower panel) of the two replicas of populations Qβ_37,_ Qβ_30,_ and Qβ_43._ Each bar represents the average of three parallel determinations carried out as described in Materials and Methods. The error bars represent the standard deviation. The fitness value obtained for the ancestor population, Qβ_0,_ at each temperature is indicated by a discontinuous line. Thus, bars above this line represent fitness increases relative to the ancestor and bars below the line correspond to fitness decreases. Asterisks mean that the average fitness for a given population is significantly higher (black ones) or lower (red ones) than the fitness value of population Qβ_0_ (*P*<0.05, Student' *t* test).

A heterogeneous population of bacteriophage Qβ (Qβ_0_), which had previously been adapted to replicate at 37°C (see Materials and Methods) was propagated during 15 serial transfers at 3 different temperatures (30°C, 37°C, and 43°C). All experiments were carried out in duplicate and the populations obtained at the last transfer were denoted Qβ_30_, Qβ_37_, and Qβ_43_ respectively (Fig. 1). Fitness values were determined at the three temperatures assayed for both the ancestor population and the evolved populations. Population Qβ_0_ had the highest fitness value at 37°C (20.50±0.19), which agrees with the optimal growth temperature of the bacterial host, an intermediate value at 30°C (17.92±1.26), and the lowest value at 43°C (9.80±0.58). Therefore, replication at 43°C was the selective pressure which produced a strongest decrease in the virus growth rate. All evolved populations experienced small, although significant, fitness gains at 37°C relative to the ancestor population (*P*<0.05, Student'*t* test; Fig. 3, upper panel). There were no significant differences among populations that could be attributable to the temperature of evolution (nested ANOVA 37°C: *F*_2,3_ = 2.0, *P*>0.05). The fact that populations propagated at 30°C and 43°C also gained fitness at 37°C probably indicates that population Qβ_0_ was not well adapted to the propagation conditions used in the adaptation experiment described in Fig. 1, and further optimization was still possible, independently of the temperature at which the virus was propagated. The generalized fitness gains at 37°C also imply that evolution at 30°C and 43°C does not have strong fitness costs on the replication of the virus at 37°C. In contrast to these results, fitness values determined at 30°C did not increase relative to the ancestor population in any of the evolved populations (Fig. 3, medium panel), and fitness at 43°C only increased significantly (*P*<0.05, Student'*t* test) in the populations propagated at this temperature (Fig. 3, lower panel). Fitness determinations at both 30°C and 43°C were significantly affected by the temperature at which populations had evolved (nested ANOVA 30°C: *F*_2,3_ = 25.5, *P*<0.025; nested ANOVA 43°C: *F*_2,3_ = 10.8, *P*<0.05). The two populations evolved at 43°C had significantly lower fitness at 30°C than population Qβ_0_ (*P*<0.05, Student'*t* test), suggesting the existence of a fitness trade-off between adaptation to 43°C and replication at 30°C. Although evolution at 43°C was carried out by using larger culture volumes (10 ml) than evolution at 30°C or 37°C (1 ml), the fitness gains attained at 43°C (observed in an assay performed in 1 ml volume) indicate that the culture volume is not confounding the interpretation of the results.

### Evolution of bacteriophage Qβ at deterministically fluctuating temperatures

**Figure 4:**
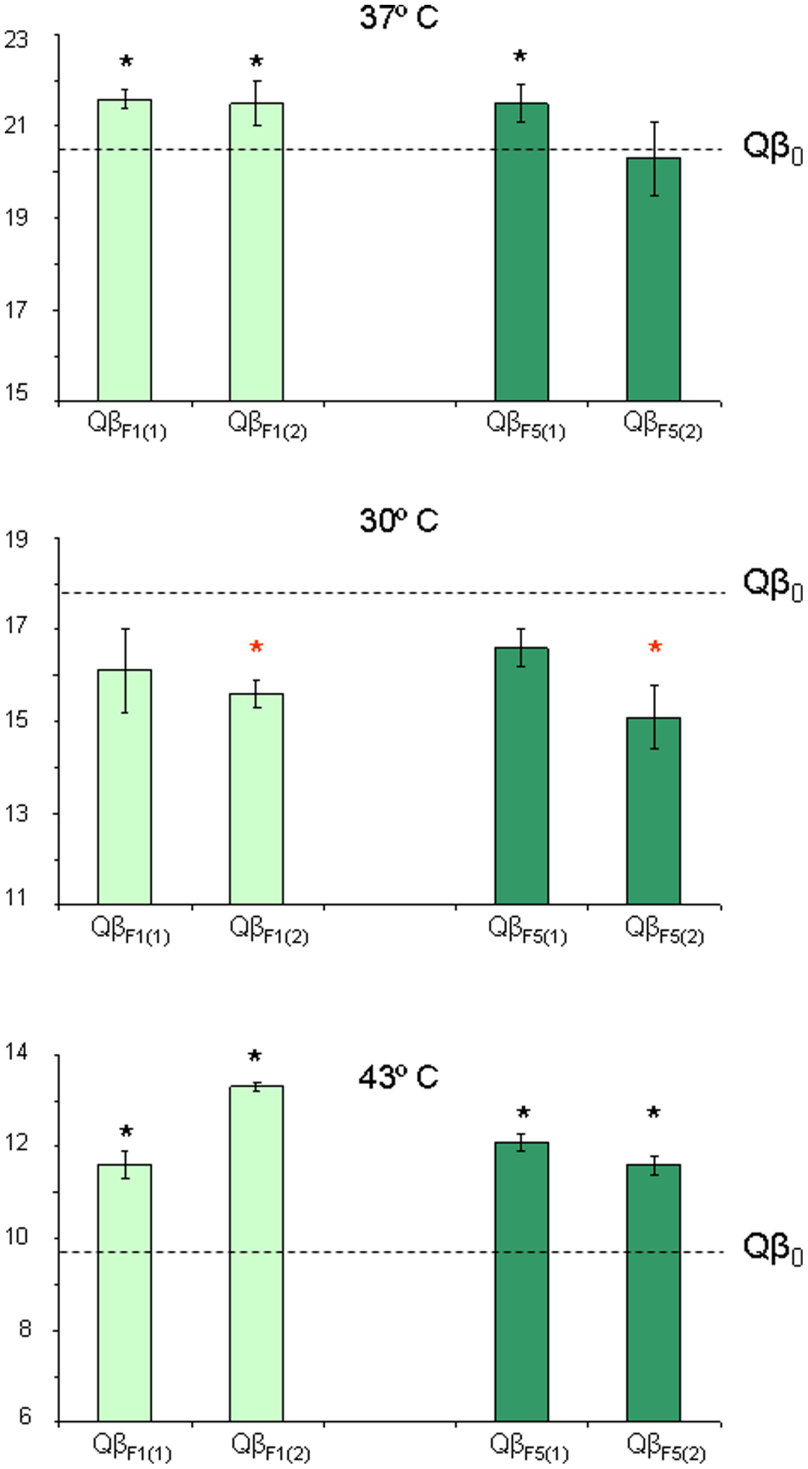
Fitness values of bacteriophage Qβ populations evolved at fluctuating temperatures. Fitness values at 37°C (upper panel), 30°C (medium panel), and 43°C (lower panel) of the two replicas of populations Qβ_F1,_ and Qβ_F5._ Each bar represents the average of three parallel determinations carried out as described in Materials and Methods. The error bars represent the standard deviation. The fitness value obtained for the ancestor population Qβ_0_ at each temperature is indicated by a discontinuous line. Thus, bars above this line represent fitness increases relative to the ancestor and bars below the line correspond to fitness decreases. Asterisks mean that the average fitness for a given population is significantly higher (black ones) or lower (red ones) than the fitness value of population Qβ_0_ (*P*<0.05, Student' *t* test).

Population Qβ_0_ was also evolved at deterministically fluctuating temperatures that alternated 37°C, 43°C, and 30°C in that order, with the temperature change occurring either every transfer (populations Qβ_F1_) or every five transfers (populations Qβ_F5_) (Fig. 1). Determination of the fitness values of populations obtained at transfer number 15 allowed us to determine whether the fluctuating conditions assayed are compatible with adaptation. The results obtained (Fig. 4) show that both Qβ_F1_ and Qβ_F5_ resembled populations Qβ_43_. They presented small though significant fitness gains at 37°C (with the only exception of Qβ_F5(2)_), fitness losses at 30°C (although they were significant only in one of the replicated populations), and significant fitness gains at 43°C. In consonance with these observations, a two-way ANOVA showed that there was no significant interaction between the temperature of evolution and the temperature of the fitness assay when populations Qβ_F1_ and Qβ_F5_ were compared to populations Qβ_43_ (*F*_4,9_ = 1.1, *P* = 0.417), whereas both factors interacted significantly when the same populations were compared to Qβ_37_ (*F*_4,9_ = 6.0, *P* = 0.013) or Qβ_30_ (*F*_4,9_ = 7.1, *P* = 0.007). It is remarkable that the fitness trade-off shown at 30°C by the populations that evolved at 43°C (Fig. 3) did not preclude adaptation to 43°C when the virus alternated these temperatures during its propagation. Evolution of bacteriophage Qβ at fluctuating temperatures shows a clear example in which the most stringent selective pressure drives adaptation. In general, fitness gains at 43°C were higher for populations evolved constantly at this temperature than for those that evolved under fluctuating temperature conditions. This could have been expected, since the former were adapting to 43°C for a larger number of transfers.

### Analysis of consensus sequences

**Figure 5:**
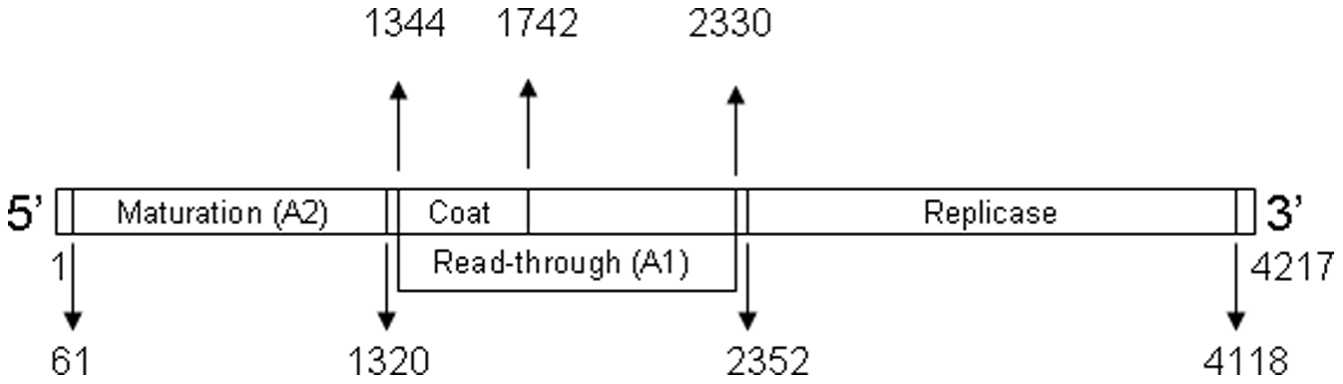
Genetic map of bacteriophage Qβ. The map shows the genes for the 4 proteins encoded by the virus [A2 (from nt 61 to nt 1320), coat (from nt 1344 to nt 1742), A1 (from nt 1344 to nt 2330, and replicase (from nt 2352 to nt 4118)], and the non-translated regions. The protein A1 synthesizes when the stop codon of the coat protein is read through by a trp-tRNA, giving rise to an elongated protein that incorporates at low amount in the capsid.

To identify the genomic substitutions potentially responsible for the observed fitness changes we determined the consensus sequences of all evolved populations and compared them to that of population Qβ_0_. To make a more thorough analysis, the mutations, either fixed or polymorphic, detected in each population were visually inspected in the chromatograms of the remaining populations to evaluate their presence as polymorphisms not recognized by the sequence analysis programs. In total we detected 42 mutations, 35 of which were present as polymorphisms (Tables 1 and 2). There were 17 different substitutions, 8 of which were present in single populations whereas the rest were repeated in at least two populations (Table 1). The total amount of repeated mutations was 84%. The percentage of coincident changes between replicated populations was higher for populations evolved under constant conditions (67% for Qβ_37_, 60% for Qβ_30_, and 43% for Qβ_43_) than for those evolved under fluctuating conditions (29% for Qβ_F1_ and 11% for Qβ_F5_). When populations evolved under different conditions were compared, the percentages of coincident changes were 60% for Qβ_37_ with Qβ_30,_ 11% for Qβ_37_ with Qβ_43_, and 9% for Qβ_30_ with Qβ_43_. In good agreement with the results obtained in the fitness assays, fluctuating populations showed a higher amount of coincident changes with populations evolved at 43°C (56% Qβ_F1_ and 33% Qβ_F5_) than with populations evolved at 37°C (25% Qβ_F1_ and 9% Qβ_F5_) or at 30°C (20% Qβ_F1_ and 8% Qβ_F5_). A high amount of mutations (76%) were non synonymous. The ratio dn/ds for the whole set of mutations was 1.1, which increased to 1.7 when the mutations represented in several populations were the only considered. The distribution of the substitutions along the Qβ genome (Table 2) shows a significant accumulation (*P*<0.025, chi squared test, 3 d.f.) in the region of the A1 protein that does not overlap with the coat protein (Fig. 5).

**Table 1:** Genomic substitutions present in the consensus sequence of the populations obtained upon evolution of Qβ_0_ at constant and deterministically fluctuating temperatures.

Substitution	Protein^1^	Amino acid change^2^	Repetitions^3^
U920A	A2	Phe286Tyr	1
A1088G	A2	Asp342Gly	3
U1198C	A2		1
U1763C	A1		1
G1773A	A1	Gly143Arg	2
C1806U	A1	Pro154Ser	4
G1817A	A1		3
A1823G	A1		1
A1930G	A1	Gln195Arg	4
U2006A	A1	Ser220Arg	1
A2187C	A1	Ser281Arg	7
U2297C	A1		1
U2776C	Rep	Val141Al	3
U3402C	Rep	Ser350Pro	6
U3731C	Rep		2
G3945A	Rep	Gly531Ser	1
C4031U	Rep		1

1 Protein encoded by the gene where the nucleotide substitution indicated is located.

2 Amino acid replacement produced by the nucleotide change. In the cases where no amino acid change is indicated, the substitution is synonymous.

3 Number of populations where a given substitution appears either fixed or as a polymorphism.

**Table 2:** Genomic location of the nucleotide positions mutated in the consensus sequences of the populations obtained upon evolution of Qβ_0_ at constant and deterministically fluctuating temperatures.

Population	A2			Coat	A1 (Non-overlapping region with the coat protein)									Replicase				
	920	1088	1198		1763	1773	1806	1817	1823	1930	2006	2187	2297	2776	3402	3731	3945	4031
Qβ_37(1)_										*		*						
Qβ_37(2)_							*			*		*						
Qβ_30(1)_							*			(*)						(*)	*	
Qβ_30(2)_							*			(*)		*				*		
Qβ_43(1)_		*						*				*	*		*			
Qβ_43(2)_		*				*		*						*	*			
Qβ_F1(1)_												*		*	*			
Qβ_F1(2)_						*	*	*				(*)			*			*
Qβ_F5(1)_		*			*						(*)			(*)	*			
Qβ_F5(2)_	*		*						*			(*)			*			

The asterisks indicate the presence of a mutation at the position indicated. All mutations were polymorphic at transfer number 15, except those shown in brackets which were fixed.

The analysis of the consensus sequences of the populations evolved under constant conditions shows that substitutions C1806U and A1930G were selected at 37°C and 30°C, and substitution A2187C was selected at the three temperatures (Table 2). There were also several substitutions that only appeared in the populations evolved at 30°C (U3731C and G3945A), or at 43°C (A1088G, G1773A, G1817A, U2297C, U2776C, and U3402C) (Table 2). In contrast to this, the populations evolved at 37°C did not show any specific mutations. From all these substitutions, the changes G1773A, C1806U, G1817A, A1930G, A2187C, and G3945A had previously been detected in our laboratory during the propagation of bacteriophage Qβ, suggesting that they are beneficial mutations of general effect under the culture conditions used in our lab [40], [41]. Therefore, they are good candidates to be responsible for the small fitness gains observed at 37°C in populations evolved at 37°C, 30°C, and 43°C. Substitutions exclusive to a particular temperature, and not detected during the evolution of the virus under unrelated selective pressures, probably represent adaptive mutations for that particular condition. In this way, substitution U3731C, only detected in populations Qβ_30_, is a candidate to promote adaptation to 30°C. Similarly, substitutions A1088G, U2297C, U2776C, and U3402, could contribute to the fitness gains observed at 43°C.

The comparison of the substitutions found in the consensus sequences of the populations evolved under constant and fluctuating temperatures allowed us to determine whether the similarities shown at the phenotypic level by Qβ_F1_ and Qβ_F5_ with Qβ_43_ are also displayed at the genetic level. None of the populations Qβ_F1_ and Qβ_F5_ showed the substitutions exclusive to the population evolved at 30°C. In contrast to this, all substitutions but one (U2297C) of those exclusive to the populations evolved at 43°C were present at least in one of the populations evolved under fluctuating conditions (Table 2), showing that this temperature also drives adaptation at the molecular level. One of the common mutations to populations Qβ_30_ and Qβ_37_ (C1806U) was present in one of the Qβ_F1_ populations, and the most widespread substitution, A2187C, was detected in the two Qβ_F1_ and in one Qβ_F5_ populations. Both Qβ_F1_ and Qβ_F5_ also have exclusive mutations. Substitution C4031U was unique to one of the Qβ_F1_ populations, whereas substitutions U920A, U1198C, U1763C, A1823G, and U2006A were only detected in the Qβ_F5_ populations (Table 2). Again, some of these substitutions (U1763C and A1823G) had previously been detected during the propagation of bacteriophage Qβ under different selective pressures in our lab [41].

### Adaptation to 43°C of populations differing in the pre-existent genetic diversity

The high number of convergent mutations detected in the Qβ populations analyzed in this work (Tables 1 and 2) could be a consequence of their previous presence as minority variants in the mutant spectrum of the ancestor population Qβ_0_. We investigated whether the same set of mutations repetitively detected in populations exposed to 43°C would be selected when evolution starts with Qβ populations differing in their pre-existent genetic diversity. To this end, we prepared a clonal population of the virus (cQβ) and adapted it to replicate at 43°C either directly or after a pre-adaptation step to 37°C (Fig. 2). Upon propagation of cQβ at 37°C, both replicated populations cQβ_37(1)_ and _(2)_ fixed substitution A2187C, which was the most frequent in our previous experiments (Tables 1 and 2). There were no signs of polymorphism at any of the other mutated positions previously identified (Tables 1 and 2). Evolution of cQβ_37(1)_ at 43°C rendered populations cQβ_37-43(1)_ and _(2)_: they maintained substitution A2187C and acquired the polymorphic substitutions A1088G, G1817A, U2776C, and U3402C, all exclusive to populations evolved at 43°C (Table 2). Although this experiment shows the selection of a common set of mutations when populations differing in their pre-existent genetic diversity (Qβ_0_ and cQβ_37(1)_) were exposed to the same selective pressure (43°C), we cannot exclude the possibility that those substitutions were generated during the propagation of cQβ at 37°C, and, thus, were already present, although at undetectable frequency by the sequencing methods employed, in the mutant spectrum of population cQβ_37(1)_. To determine whether the same mutations could also be selected when all the genetic diversity is generated *de novo* during adaptation, we sequenced the two replicated populations cQβ_43(1)_ and cQβ_43(2)_ generated from cQβ directly evolved to 43°C (Fig. 2). We detected the same set of polymorphic substitutions (A1088G, U2776C, U3402C, and U3784C) in both of them, confirming that substitutions A1088G, U2776C, and U3402C represent an adaptive pathway to high temperature that is accessible from different initial conditions. Substitution U3784C, which was not selected during the adaptation of Qβ_0_ at 43°C, could also be involved in adaptation to 43°C or be a substitution that increases the growth rate by a mechanism independent of temperature.

## Discussion

Living organisms have to cope with frequent environmental changes that limit their survival if they are not able to adapt to the new conditions. When the selective pressures remain constant for a long time, populations usually evolve to perform optimally under those conditions, even if that entails a decrease of fitness in alternative environments [1]–[7]. On the contrary, when the selective pressures change frequently, populations do not have the time necessary to achieve the best adaptive solution for each condition [42]. The situation gets further complicated when mutations selected in a particular environment have deleterious effects in a different environment encountered by the population. The study of the evolutionary pathways followed by populations adapting to constant versus fluctuating environments is particularly interesting in the case of RNA viruses whose great capacity to adapt to new selective pressures hamper most antiviral treatments [43]–[45]. We have addressed this question by exposing an RNA virus, the bacteriophage Qβ to three constant environments that differed in the temperature (37°C, 43°C, and 30°C), and to two deterministically fluctuating environments in which the temperature alternated among 37°C, 43°C, and 30°C with two patterns of change.

### Propagation of bacteriophage Qβ at different constant temperatures does not result in a common pattern of adaptation

We found that propagation of bacteriophage Qβ at any of the temperatures assayed increased the performance of the virus at 37°C, indicating that either the fitness effects of some of the mutations selected in lineages exposed to different treatments correlate across different temperatures or selection is acting on viral traits unrelated to this character. Since in all cases the virus was propagated according to a similar transmission regime, fitness increases at 37°C can arise as a consequence of adaptation to the culture conditions used. The coincidence of several mutations in populations evolved under different temperature conditions indicates that the common behaviour observed at 37°C has a genetic basis. The fact that several of those coincident mutations had also been detected in previous experiments in which bacteriophage Qβ was propagated in our lab under unrelated selective pressures also agrees with a general beneficial fitness effect on the adaptation of the virus to the culture conditions used [40], [41]. Fitness gains at a temperature different from that at which the virus was propagated have also been reported for vesicular stomatitis virus (VSV) adapted to 29°C and 37°C [33]. Another study that compared the adaptation to 44°C in the related viruses PhiX174 and G4 showed the existence of a fitness cost at 37°C in PhiX174 but not in G4 [46]. In our study, the general fitness gains observed at 37°C had no counterpart at 30°C or 43°C. In fact none of our populations increased fitness at 30°C, and only populations Qβ_43_ improved their performance at 43°C. This result suggests that the inhibition of virus growth that takes place at 30°C and 43°C masks the beneficial effect of the mutations that provide selective advantages unrelated to temperature. The observed difficulties for adaptation to 30°C agree with the hypothesis that argues that the rate-depressing effects of low temperature on biochemical reactions are difficult to overcome by genetic changes [47].

Populations adapted to 43°C performed worse at 30°C than the ancestor population, suggesting that some of the mutations responsible for adaptation to high temperature are deleterious when this parameter decreases below the optimal value. A possible explanation for this trade-off is that adaptation to high temperature selects for mutations that render more thermodynamically stable proteins, whose performance worsens when the temperature is lowered [48]–[51]. The main argument for the trade-off between function and stability lies in the belief that catalytic proteins must not be too stable to allow for function-related flexibility [52], a hypothesis supported by the fact that the stability of real proteins is not too high. Also there could be a trade-off between growth rate and survival, particularly at high temperatures, a possibility that deserves to be further analyzed.

### Bacteriophage Qβ populations evolved under deterministically fluctuating temperatures perform similarly to populations propagated under the most restrictive condition

The fitness trade-off between adaptation to 43°C and virus performance at 30°C could pose a conflict when the virus has to thrive in environments in which the two temperatures alternate. However, populations evolved under fluctuating conditions showed striking similarities with populations evolved at 43°C. This result agrees with the selection of an evolutionary pathway that favours adaptation to the most restrictive condition, in this case 43°C, even if that means that the virus decreases its performance under other less stringent selective pressure (30°C). This pathway is probably favoured because the fitness cost at 30°C is lower than the growth inhibition that takes place at 43°C in non-adapted viruses. It is striking that we observe the same pattern of fitness across environments in the populations Qβ_F1_ and Qβ_F5_, which alternated temperatures differently. It could have been expected that mutations responsible for fitness gains at 43°C had been eliminated by negative selection during virus replication at 30°C, particularly in the population Qβ_F5_.

Our results differ from those obtained by Alto et al. during the adaptation of VSV to deterministically changing temperatures (29°C alternating with 37°C), where a generalist population with increased performance at each temperature was selected [33]. These results could have been facilitated by the absence in VSV of fitness trade-offs across the two temperatures assayed. Studies carried out with viruses challenged to alternate between different hosts sometimes also show equal or superior fitness gains on each host compared to viruses constantly evolved in the same host [22], [24], [27]–[29], [32], [33]. These fitness gains in the generalist lineages can even occur when trade-offs across different hosts exist [22].

### The consensus sequences of the evolved bacteriophage Qβ populations show coincident mutations at different temperatures together with mutations exclusive to a particular condition

Most of the mutations that we found in the consensus sequences of the evolved bacteriophage Qβ populations were polymorphisms. A possible explanation is that not enough generations were allowed for mutations to reach fixation. An alternative interpretation could be that different beneficial mutations occurred in different lineages and competed with each other, such that interference would delay their fixation [53]–[55]. Antagonistic epistatic interactions among different beneficial mutations could also contribute to prolong the time that mutations remain as polymorphisms, especially if some of them cannot coexist in the same genome. This kind of interactions has been described in different viruses [56]–[58], and was shown to be responsible for the prolonged co-existence of two beneficial mutations during the adaptation of bacteriophage Qβ to a mutagen [41]. Investigating this question would require an analysis of the distribution of mutations in the individual components of the mutant spectra of the evolved Qβ populations. Propagation of the virus for a larger number of transfers would also help to determine whether a defined consensus sequence can be obtained.

The analysis of the consensus sequences of the evolved populations show a high number of coincident mutations. This fact, together with the high value of the ratio dn/ds calculated for the repeated mutations, is indicative of the action of natural selection. In the case of RNA, selection may also act at the level of synonymous mutations, whose fitness effect might appear through modifications of the RNA secondary and tertiary structures or by affecting genomic domains required for interaction with substrates. The high degree of convergent evolution here uncovered is similar to that found in other studies carried out with viruses adapting to a constant selective pressure [59]–[61].

In addition to the mutations repeated in populations propagated under different conditions, other mutations only appeared in the sequence of the virus exposed to a particular temperature. Some of these mutations also coincided in replicated populations, and thus are likely responsible for adaptation to a given temperature. Specifically, substitution U3731C could promote adaptation to 30°C, although this effect was not reflected in fitness increases at 30°C, which could mean that it acts on a trait with no influence on the growth rate. The other substitution exclusive to 30°C but only present in one of the replicated populations was G3945A, which was previously demonstrated to have a general beneficial fitness effect in experiments carried out in both our lab [41] and in others [62]. In the same way, substitutions A1088G, G1817A, U2776C, and U3402C, exclusive to populations evolved at 43°C, could be responsible for the fitness gains observed at this temperature. There were no mutations exclusive to 37°C, which agrees with the fact that the ancestor population Qβ_0_ had previously been propagated at 37°C and, therefore, the supply of beneficial mutations specific to this temperature could be exhausted. To establish unambiguous correspondences between particular mutations and their fitness effect, it would be desirable to build single site-directed mutants and determine their fitness at the temperature of interest, either in competition with the wild-type virus or through the estimation of the growth rate.

Populations evolved under fluctuating conditions, which were phenotypically similar to the constant ones evolved at 43°C, also resembled those populations at the genetic level. They presented some of the common mutations selected at different constant temperatures, did not have any of the mutations exclusive of 30°C, and showed most of the mutations exclusive of the populations evolved at 43°C, confirming their implication in the adaptation of bacteriophage Qβ to high temperature.

### Evolution at 43°C of bacteriophage Qβ populations differing in their pre-existent genetic diversity leads to the selection of a common set of mutations

The high degree of parallel evolution shown by the populations analyzed in this work could be due to the fact that the ancestor population was heterogeneous, and, thus, it could contain in its mutant spectrum some of the mutations that consistently appeared during evolution in different lineages. To investigate this possibility we compared the mutations selected during adaptation to 43°C in populations that differed in their pre-existent genetic diversity. We observed that a clonal population and a heterogeneous population, obtained upon transmission at 37°C of the clonal population, coincided in the selection of the polymorphic substitutions A1088G, U2776C, and U3402C, which had previously been identified during the adaptation of population Qβ_0_ to 43°C. The result indicates that there are few pathways for adaptation of bacteriophage Qβ to high temperature. The population adapted directly to 43°C showed the additional selection of U3784C, which could also provide selective advantages. Pre-adaptation of the clonal population of bacteriophage Qβ to 37°C led to the fixation of a single substitution, A2187C, in two replicate experiments. Therefore, at least the most repeated substitution across all evolutionary lineages analyzed in this work could also be generated *de novo* during propagation of cQβ at 37°C. We did not detect any of the other substitutions to which we had attributed general beneficial effects, meaning that they were probably present in the mutant spectrum of population Qβ_0_ and reached high frequency upon its propagation.

### Molecular mechanism of action of the mutations fixed during adaptation

Most mutations selected in lineages evolved at different temperatures were placed in the A1 protein and corresponded to the replacement of different amino acids by arginine residues. The A1 protein incorporates in 3–10 copies per virion and is essential for producing infectious virus particles, although its precise function remains unknown. It has recently been suggested that some domains of the A1 protein can be involved in protein interactions that would be necessary to reach the virus fivefold and threefold symmetry axes [63]. Therefore, our results could mean that structural constraints on the virus capsid limit replication under the transmission regime used, and selection of the A1 mutants helps to overcome those constraints. Some of the lineages evolved at 43°C showed the selection of substitution G1817A, also placed in the A1 protein. Although this is a synonymous substitution, we cannot discard that it has some beneficial effect either in stabilizing the viral RNA or in altering somehow its interaction with other molecules.

Adaptation to 43°C was associated to the selection of substitution A1088G (Asp342Gly) in the A2 protein, which is involved in the recognition of the conjugative pilus as receptor in F+ bacteria, the escort of the genomic RNA into the host cytoplasm, and the lysis of the bacteria to release the virus progeny. High temperatures might impair any of these processes, which would be restored, at least in part, in the mutant. It is interesting that during the evolution of bacteriophage phi6 in a novel temperature environment where heat shock imposed extreme virus mortality, a single amino acid substitution in the viral lysis protein was also selected [51].

The replicase substitutions selected at 43°C, U2776C, U3402C, and U3784C rendered the non synonymous changes Val141Ala, Ser350Pro, and Ile477Thr respectively. Ser350 is placed in the palm domain of the Qβ replicase, whereas Val141 is placed in the fingers domain, and Ile477 is in thumb domain [64], [65]. The three residues are not conserved among members of the virus family Leviviridae, which suggests that they do not form part of the active center of the Qβ replicase. Moreover, the related viruses MX1 and M11 contain proline instead of serine at position 350, the viruses NL95, GA, MS2, and FR contain alanine instead of valine at position 141, and threonine instead of isoleucine is found in the viruses NL95, SP, F1, and MX1 [64], [65]. The most probable explanation for the beneficial effects of these substitutions at high temperatures is that they increase the thermal stability of the replicase. The finding that some Qβ-related viruses posses in their wild type form the same amino acids as the high temperature Qβ adapted mutants suggests that what we have selected through experimental evolution in the lab was already selected in nature. Since we have shown that high temperature is the dominant force driving evolution in bacteriophage Qβ when it is exposed to fluctuations in this condition, the selection of thermostable replicases must not be an unusual fact in viruses exposed to transitory temperature increases. The determination of whether those viruses have higher tolerance to high temperatures would be very useful to check the validity of our interpretation.
